# Tracking tDCS induced grey matter changes in episodic migraine: a randomized controlled trial

**DOI:** 10.1186/s10194-021-01347-y

**Published:** 2021-11-20

**Authors:** Simon Schading, Heiko Pohl, Andreas Gantenbein, Roger Luechinger, Peter Sandor, Franz Riederer, Patrick Freund, Lars Michels

**Affiliations:** 1 Spinal Cord Injury Centre Balgrist, University Hospital Zurich, University of Zurich, Zurich, Switzerland; 2 Department of Neurology, University Hospital Zurich, Zurich, Switzerland; 3 ZURZACH Care, Bad Zurzach, Switzerland; 4 Institute for Biomedical Engineering, ETH Zurich and University of Zurich, Zurich, Switzerland; 5 Neurological Center Rosenhügel and Karl Landsteiner Institute for Epilepsy Research and Cognitive Neurology, Vienna, Austria; 6 Wellcome Centre for Human Neuroimaging, Institute of Neurology, University College London, London, UK; 7 Department of Neurophysics, Max Planck Institute for Human Cognitive and Brain Sciences, Leipzig, Germany; 8 Department of Neuroradiology, University Hospital Zurich, Frauenklinikstrasse 10, 8091 Zurich, Switzerland

**Keywords:** Transcranial direct current stimulation, Migraine, Structural alterations, Voxel-based morphometry, Brain plasticity, Structural MRI

## Abstract

**Background:**

Occipital transcranial direct current stimulation (tDCS) is an effective and safe treatment for migraine attack prevention. Structural brain alterations have been found in migraineurs in regions related to pain modulation and perception, including occipital areas. However, whether these structural alterations can be dynamically modulated through tDCS treatment is understudied.

**Objective:**

To track longitudinally grey matter volume changes in occipital areas in episodic migraineurs during and up to five months after occipital tDCS treatment in a single-blind, and sham-controlled study.

**Methods:**

24 episodic migraineurs were randomized to either receive verum or sham occipital tDCS treatment for 28 days. To investigate dynamic grey matter volume changes patients underwent structural MRI at baseline (prior to treatment), 1.5 months and 5.5 months (after completion of treatment). 31 healthy controls were scanned with the same MRI protocol. Morphometry measures assessed rate of changes over time and between groups by means of tensor-based morphometry.

**Results:**

Before treatment, migraineurs reported 5.6 monthly migraine days on average. A cross-sectional analysis revealed grey matter volume increases in the left lingual gyrus in migraineurs compared to controls. Four weeks of tDCS application led to a reduction of 1.9 migraine days/month and was paralleled by grey matter volume decreases in the left lingual gyrus in the treatment group; its extent overlapping with that seen at baseline.

**Conclusion:**

This study shows that migraineurs have increased grey matter volume in the lingual gyrus, which can be modified by tDCS. Tracking structural plasticity in migraineurs provides a potential neuroimaging biomarker for treatment monitoring.

**Trial registration:**

ClinicalTrials.gov, NCT03237754. Registered 03 August 2017 – retrospectively registered, https://clinicaltrials.gov/ct2/show/NCT03237754.

## Background

Migraine is a common relapsing headache disease with a huge socioeconomic burden. After lower back pain, it is the second most disabling condition [1]. The 2016 Global Burden of Disease Study measured a worldwide prevalence of 14.4% with 18.8% of women and 9.8% of men suffering from this disease highlighting its impact on the society [2]. Migraine attacks affect patients’ daily life considerably and have a significant negative impact on health-related quality of life in a frequency-dependent manner with higher attack frequency being associated with more severe disability [3–5]. Moreover, migraine is associated with numerous comorbidities including sleep disorders and various psychiatric diseases such as depression and anxiety disorders [6, 7]. Hence, migraine poses a substantial socioeconomic burden by both direct costs through specific migraine treatment and the associated comorbidities, and indirect costs due to work-absenteeism and loss in productivity [8].

Current treatment options for acute migraine attacks comprise mostly medication such as common non-steroidal anti-inflammatory drugs (NSAIDs) and triptans. Many patients remain unsatisfied with their medication during acute migraine attacks what highlights the importance of prevention of migraine attacks [9]. Attack prevention is multifaceted and includes lifestyle modifications, behavioral therapy, medication such as beta blockers, calcium channel blockers, antiepileptic drugs or antibodies against calcitonin gene-related peptide (CGRP) or its receptor, and neuromodulation. However, many patients do not desire prophylactic treatment with medication which is represented in relatively low adherence rates [10].

Several studies have investigated the efficiency of transcranial direct current stimulation (tDCS) in migraine prevention [11–16]. Most of them reported significant reduction in migraine days when applying tDCS to the occipital cortex, primary motor cortex, or the dorsolateral prefrontal cortex [11, 12, 14–16]. The advantages of tDCS treatment are its relatively low cost compared to other neurostimulation methods, safety and generally mild side effects [17]. tDCS acts by modifying cortical excitability through hyperpolarization or subthreshold depolarization of neurons depending on direction of current flow [18–21]. This effect can be used in migraine treatment as the brains of migraineurs exhibit altered cortical excitability and information processing over the migraine cycle [22–26].

The results from several studies indicate that additionally to having altered functionality in terms of cortical excitability, migraineurs undergo structural changes in several cortical and subcortical areas related to perception and pain processing, including the occipital cortex, when comparing them to a healthy population [27]. Moreover, longitudinal observations suggest that these alterations are dynamic over time and respond to beneficial migraine treatment [28–30].

A clinical trial, investigating the efficiency of a 28-day occipital anodal tDCS stimulation found a reduction of monthly migraine days of 2.6 days during the third month after tDCS treatment, while at earlier and later time points the tDCS and sham cohort did not differ significantly. Based on this clinical report the present study aims to investigate structural differences between episodic migraineurs and healthy controls by means of voxel-based morphometry (VBM) and at tracking these morphological alterations up to 5 months after a 28-day treatment period with occipital tDCS using tensor-based morphometry (TBM) for assessing the temporal dynamics of structural plasticity. Given the location of tDCS treatment and results from literature, we focused on structural changes in occipital cortical areas. Based on literature we hypothesized that (i) episodic migraine patients show structural alterations in occipital areas compared to a healthy control population; (ii) these alterations are dynamic over time and can be reversed by successful occipital tDCS treatment.

## Materials and methods

### Participants and exclusion/inclusion criteria

In this study, we enrolled patients between the age of 18 and 80 that had a preexisting diagnosis of “episodic migraine (EM) without aura” or “EM with and without aura” according to the International Classification of Headache Disorders, 3rd edition (ICHD-3) criteria. All headaches were diagnosed by an experienced neurologist [31]. Exclusion criteria included pregnancy, presence of a neurodegenerative disease, and contraindications against magnetic resonance imaging (MRI).

We also recruited healthy individuals as a control group who underwent the same imaging protocol as the patients.

All participants gave their informed consent and the study was approved by the local ethics committee.

### Study design

This longitudinal, single-blind, randomized and sham-controlled trial was conducted at the University Hospital Zurich [32]. The migraine patients were randomized to either receive verum or sham tDCS treatment by using a block randomization technique with block sizes of ten (five verum and five sham per block).

**Fig. 1:**
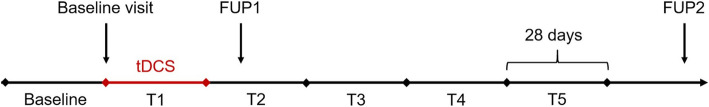
Overview of the study design. The observation period was subdivided into six 28-day periods (Baseline, T1–5). The tDCS treatment was performed during T1 and was initiated immediately after the baseline visit. FUP1 was scheduled shortly after the end of the stimulation period and FUP2 after T5. Modified from Pohl et al. [32]

During the whole study period, patients kept a headache diary and recorded the following parameters: occurrence, duration, quality, and intensity of headache attacks, as well as medication intake and accompanying features. The study period was divided into six subsequent blocks of 28 days, which will be referred to in the following as baseline period, T1, T2, T3, T4, and T5 (see Fig. 1). The baseline period served to assess patient’s migraine characteristics using the same headache diary and to validate the diagnosis of EM. During T1, directly following the baseline visit, the 28-day lasting tDCS treatment was performed. At three time points, migraine patients underwent clinical examination as well as structural MR imaging. These time points (baseline visit, FUP1, FUP2) are indicated in Fig. 1. The clinical outcome of this study is reported separately [32].

### Transcranial direct current stimulation

In this study, patients themselves applied anodal stimulation over the visual cortex by using a one-channel stimulator and standard rubber tDCS electrodes (DC-STIMULATOR PLUS, NeuroConn, Ilmenau, Germany). The active electrode (electrode size 5 × 7 cm^2^, current density 0.029 mA/cm^2^) was placed at Oz, located at the inion. A reference electrode (electrode size 10 × 10 cm^2^, current density 0.01 mA/cm^2^) was placed at Cz at the intersection between a sagittal line connecting the nasion and the inion and a coronal line between the tragus of both ears. Whereas the active electrode consisted of a more focal electrode resulting in a higher current density, a larger electrode was chosen as reference. This allowed maximizing the current density over the visual cortex while rendering the reference electrode functionally ineffective.

Patients were instructed on placing of the electrodes and handling of the device at the baseline visit.

The tDCS treatment was performed daily for 20 min during the 28-day T1 period (compare Fig. 1). The verum tDCS consisted of applying 1 mA over the 20-min session, while during sham stimulation 1 mA intensity was maintained only for 30 s with intermittent impedance check in the remaining 1170 s.

### MRI measurements

We scanned patients with a 3 Tesla Philips Ingenia scanner (Philips Healthcare, Best, The﻿ Netherlands) with a 32-channel receive-only head coil at the Neuroimaging Center of the University Hospital Zurich. Apart from other sequences, a 3D T1-weighted magnetization prepared rapid gradient echo (MPRAGE) sequence was acquired for each subject. Scanning parameters were as follows: 160 slices, repetition time: 8.1 ms, echo time: 3.7 ms, flip angle: 8°, voxel dimensions: 1 × 1 × 1 mm, field of view: 240 × 240 mm^2^, scan time: 4:32 min. An experienced neuroradiologist examined all structural images for the presence of any brain abnormalities.

This imaging protocol was conducted for each patient at three time points, baseline and two follow-up sessions. The first follow-up measurement was scheduled at 1.5 months following the baseline scan and the second measurement approximately 7 months after baseline (compare Fig. 1).

Controls were scanned with the same imaging protocol at baseline and one follow-up session at 1.5 months post-baseline.

### MRI processing

A total of 3 structural T1w images were acquired for each patient and 2 images for healthy controls on which voxel based morphometry methods were applied for estimation of grey matter (GM) and white matter (WM) changes in the brain [33].

The volumetric analysis was subdivided into two separate analyses, one cross-sectional comparison at baseline between healthy subjects and migraine patients and one longitudinal analysis of migraine patients for the time points baseline, FUP1 and FUP2, for which slightly different imaging processing pipelines were employed.

For the assessment of volumetric differences between EM patients and healthy controls, we used voxel-based morphometry within SPM12 (University College London, London, UK). First, the baseline T1w-MPRAGE images of each subject were segmented into GM, WM and cerebrospinal fluid (CSF) by using unified segmentation [34] which produced three probabilistic maps of these respective tissue types. This step was followed by spatial normalization of these probabilistic maps into standard Montreal Neurological Institute (MNI) space with a diffeomorphic Anatomical Registration using Exponentiated Lie algebra (DARTEL) algorithm [35] and application of smoothing with an isotropic Gaussian kernel of 3 mm full width at half maximum (FWHM).

For assessing regional longitudinal volumetric changes in migraine patients, we used TBM inside the SPM12 framework. First, the T1w MR images of the three time points were longitudinally co-registered, based on a registering of each imaging volume to a subject-specific average map. This step includes non-linear and rigid-body registration with corrections for intensity bias artifacts. The results of this step were subject-specific average maps representing a midpoint image, corresponding deformation fields and Jacobian Determinant Maps from each time point to this average map. This average map was segmented into the different tissue components using the same unified segmentation as described above. By applying the Jacobian Determinant Maps on the midpoint image, the respective tissue probability maps of each separate time point were obtained. For improved normalization into standard MNI space, the DARTEL algorithm was applied to the midpoint images and the produced deformation fields were used to register each individual time point to MNI space. Finally, an isotropic smoothing kernel of 3 mm FWHM was applied.

The total intracranial volume (TIV), used as covariate in the statistical models, was calculated as the sum of brain GM, WM and CSF volumes defined by a cut-off at the lowest slice including the cerebellum.

### Statistical analysis

Descriptive analysis of demographic data was performed by using parametric and non-parametric statistics in STATA 17.0 (StataCorp LLC, College Station, TX, USA) and included two-sided t-tests, Wilcoxon rank-sum test, Kruskal-Wallis test and Fisher’s exact test.

To assess volumetric differences in GM and WM of the brain and between patients and controls at baseline general linear models were fitted with the covariates TIV, sex and age inside the SPM12 framework. Then, voxel-wise two-sample T-tests were conducted for comparison of volumetric increases or decreases between patients and controls. In order to assess the effect of the presence of aura on these observed volumetric changes we performed one-way ANOVA at baseline in the identified regions including the groups “Controls”, “EM with aura”, “EM without aura”. Furthermore, we assessed whether the observed structural alterations correlated with clinical factors representing disease severity such as monthly migraine days and migraine intensity, and the total number of years that patients are suffering from migraine. This correlation analysis was implemented with a similar model in SPM12 by adding the respective clinical factor as an additional covariate. Significance threshold was set at *p* < 0.001 uncorrected for multiple comparison at voxel-level with a cluster extent (CE) threshold of 20 contiguous voxels for all analyses. Only significant results with *p* < 0.05, corrected for family-wise error (FWE) at the cluster level using Random Field Theory are considered.

For the assessment of longitudinal volumetric changes, we used the SPM Sandwich Estimator Toolbox (SwE) for Longitudinal & Repeated Measures Data, which is based on a marginal model where the expected variability is described as a function of predictors (defined in the design matrix) and additionally accounts for correlations due to repeated measurements and unexplained variations across individuals. It has the following form for subject *i*:

$$ {y}_i={X}_i\beta +{\epsilon_i}^{\ast } $$

where *y*_*i*_ represents the tissue volumes at multiple timepoints, *X*_*i*_ denotes the design matrix and *ϵ*_*i*_^∗^ the random effects modelled by individual marginal error terms with between-subject variance components with mean 0 [36]. The design matrix *X*_*i*_ consisted of the predictors intercept, time and time^2^ as well as the covariates age, sex, TIV, number of migraine days during baseline period, number of stimulation days, the Depression subscale of the Hospital Anxiety and Depression Scale (HADS-D), and the presence of aura. We chose to introduce a quadratic term due to the observed quadratic behavior of the clinical effect reported by Pohl et al. [32] and the assumption that potential volumetric changes would behave likewise. The HADS-D score was included because there is evidence that depression alters cortical excitability and hence might influence the efficiency of tDCS treatment [37–40]. We tested for significant differences in the quadratic component between tDCS and sham patients with a cluster inference threshold of *p* < 0.001 and a CE threshold of 20 contiguous voxels. Results with q < 0.05 (FDR-corrected) were considered as significant.

A correlation analysis was additionally performed to assess whether the GM changes are associated with the clinical effect of tDCS therapy represented by changes in the number of monthly migraine days. This correlation analysis was conducted by using a similar model within the SwE framework and adding the number of migraine days at baseline, T2 and T5 as an additional covariate.

## Results

### Demographics, baseline characteristics, and clinical outcome

We enrolled 31 healthy controls and 24 episodic migraineurs in this study. One control was excluded from further analysis due to severe artifacts on MRI. The baseline characteristics of the control and patient cohorts including subdivision of patients into migraineurs with (MwA) and without aura (MwoA) were not different in age, sex, number of migraine days, migraine intensity and duration of migraine disease (Table 1).

**Table 1:** Baseline characteristics of controls, episodic migraineurs with aura and episodic migraineurs without aura

	Controls (***N*** = 30)	MwA (***N*** = 15)	MwoA (***N*** = 9)	***p*** value
Age, years	32.2 ± 10.3	37.0 ± 12.9	39.1 ± 12.0	0.212
Female Sex, no. (%)	25 (83.3)	15 (100)	8 (88.9)	0.225
Average number of migraine days during baseline period		5.6 ± 2.9	5.7 ± 2.2	0.933
Average migraine intensity during baseline period		6.0 ± 1.6	5.6 ± 1.6	0.514
Disease duration, years		18.5 ± 10.9	21.5 ± 13.1	0.603

*MwA* Migraineurs with aura; *MwoA* Migraineurs without aura; two-sided t-tests were applied for comparison of number of migraine days, migraine intensity and duration of migraine disease between MwA and MwoA; Kruskall-Wallis test was used for comparison of age and Fisher’s exact test for sex between Controls, MwA, and MwoA.

Likewise, in both randomized groups (tDCS and sham), migraine patients did not differ with respect to age, sex, average number of migraine days, average migraine intensity, disease duration, patients with aura, number of stimulation days, HADS-D score and timing of the follow-up measurements (Table 2). However, the timing of the second follow-up measurement displayed a relatively high variation ranging from 98 days to 373 days post-baseline.

**Table 2:** Baseline characteristics of episodic migraineurs randomized into tDCS and sham subgroups

	tDCS (***N*** = 11)	Sham (***N*** = 13)	***p*** value
Age, years	41.0 ± 14.8	35.1 ± 9.7	0.251
Female Sex, no. (%)	10 (90.9)	13 (100)	0.458
Average number of migraine days during baseline period	4.7 ± 2.2	6.4 ± 2.7	0.118
Average migraine intensity during baseline period	6.3 ± 1.2	5.4 ± 1.8	0.199
Disease duration, years	22.0 ± 12.4	18.5 ± 11.6	0.546
Aura, no. (%)	7 (63.6)	8 (61.5)	1.000
Number of stimulation days	30.1 ± 2.9	28.8 ± 1.9	0.147
HADS-D score during baseline period	3.8 ± 2.2	4.1 ± 3.4	0.829
FUP1, days	48.7 ± 6.9	50.2 ± 8.5	0.644
FUP2, days	161.5 ± 52.5	174.2 ± 69.5	0.352

*FUP1* first follow-up; *FUP2* second follow-up; *HADS-D* depression subscale of the Hospital Anxiety and Depression Scale; two-sided t-tests were applied for comparison of continuous variables; where the criterion of normal distribution was not met (FUP2, number of stimulation days), Wilcoxon rank-sum test was used; Fisher’s exact test was performed for comparison of sex and aura between tDCS and sham. Modified from Pohl et al. [32].

The clinical outcome of this study is reported separately [32]. A 28-day treatment with occipital tDCS led to a mean reduction of 1.9 migraine days/month compared to the control group. At the beginning and the end of the observation period no antimigraine effect of tDCS treatment was detected, but the effect gradually developed over time and reached its maximum at four months after the initiation of the treatment.

### Cross-sectional VBM analysis

**Fig. 2:**
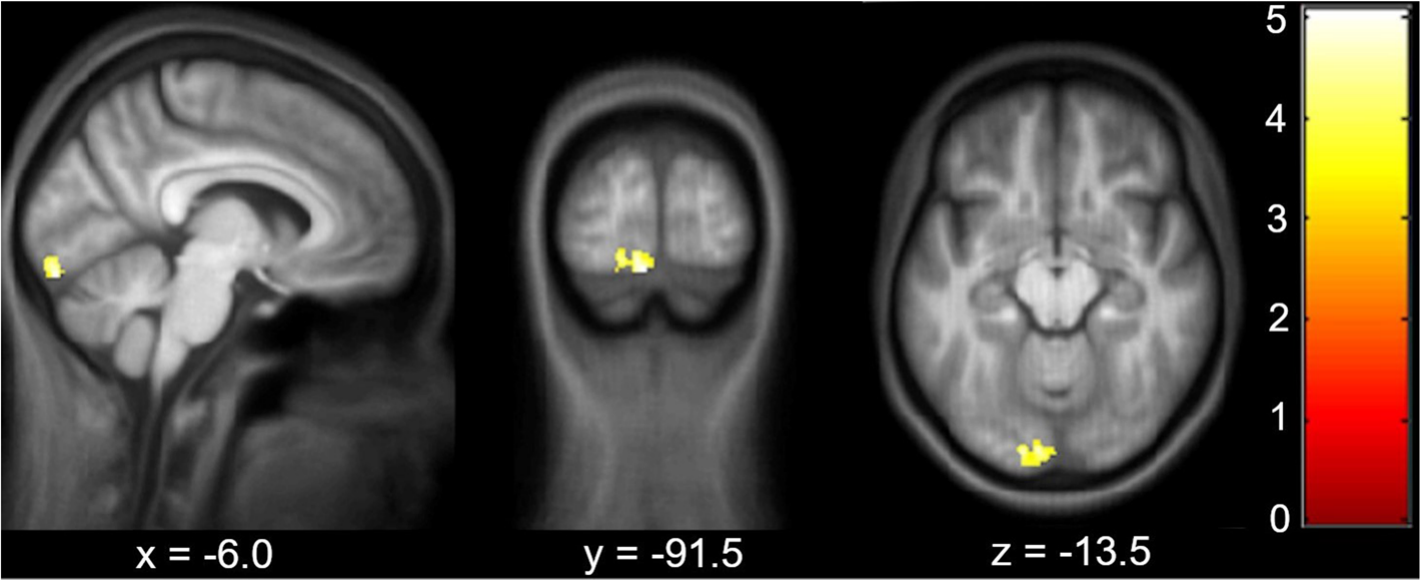
Significant volumetric differences between migraine patients and healthy controls at baseline. Overlay of statistical parametric maps (uncorrected *p* < 0.001) shows increased cortical volume in patients versus controls in the left lingual gyrus. The color bar indicates the t-score

When comparing healthy controls with migraineurs at baseline, migraineurs showed significantly increased GM volume in the left lingual gyrus (z-score = 4.53, x = − 6, y = − 92, z = − 17, *p* = 0.003, CE = 228) (see Fig. 2). There were no significant differences in WM volume. The subgroup analysis did not show any significant volumetric differences between migraineurs with aura and migraineurs without aura in this identified region.

### Correlation of structural alterations with clinical parameters

The correlations of structural brain changes with the following clinical parameters were assessed: monthly migraine days, migraine severity and disease duration. We did not detect any significant associations between structural alterations and the clinical factors in this patient cohort.

### Longitudinal TBM analysis

**Fig. 3:**
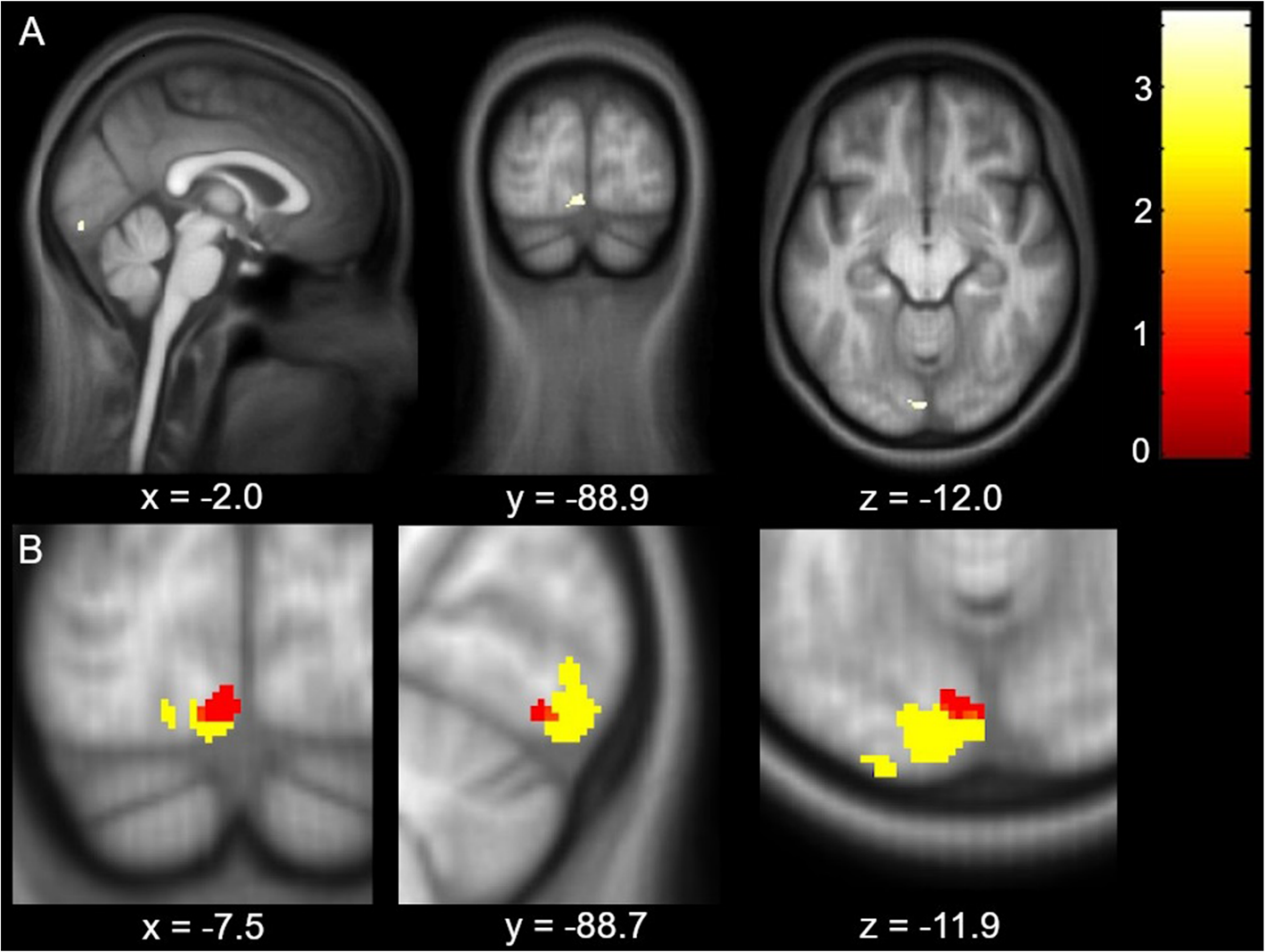
Significant quadratic differences between verum and sham tDCS group revealed by TBM. Overlay of statistical parametric maps (uncorrected p < 0.001) shows a significant quadratic difference in the left lingual gyrus (**A**). The color bar indicates the t-score. Comparison of the statistical parametric maps (uncorrected *p* < 0.005, for illustrative purposes) of the cross-sectional cluster (in yellow) and the longitudinal cluster (in red) reveals that the regions of both clusters in the left lingual gyrus overlap (**B**)

Investigating dynamic volumetric changes over the observation period of approximately 170 days after the baseline visit we found a significant difference in the rate of quadratic GM change in the left lingual gyrus between the sham and verum tDCS group (z-score = 3.62, x = − 2, y = − 90, z = − 12, q = 0.039, CE = 23) (see Fig. 3A). Note, this cluster of dynamic GM volume change overlaps with the identified cluster in the cross-sectional analysis at baseline where migraineurs possessed increased cortical volume when compared to healthy controls as indicated in Fig. 3B.

**Fig. 4:**
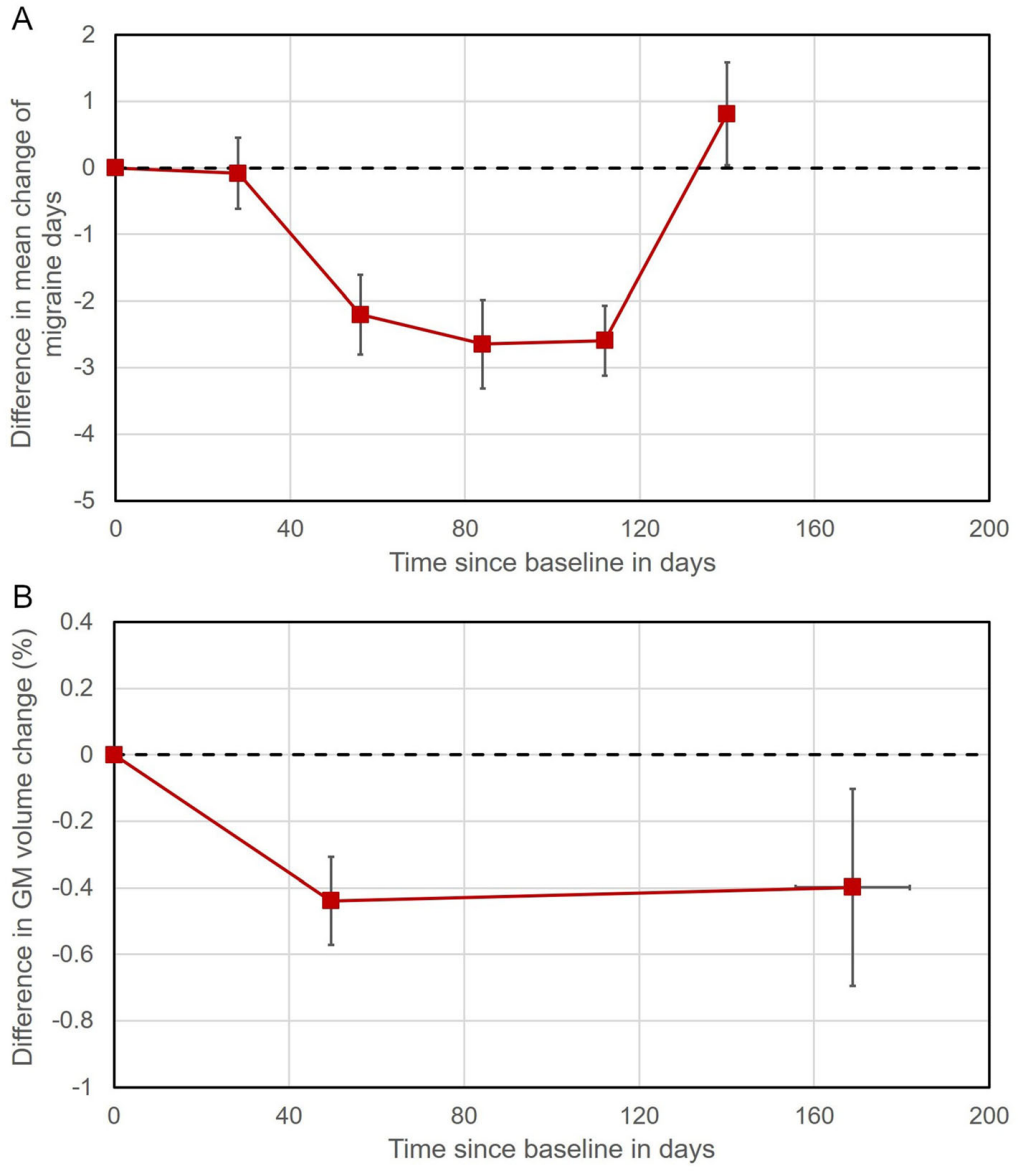
Clinical and structural changes following tDCS treatment. Change in monthly migraine days depicted as difference in days between verum and sham tDCS group (**A**). Change in GM volume depicted as difference in percent between verum and sham tDCS group (**B**). Negative values represent less migraine days, respectively lower GM volume in the verum tDCS group compared to the sham tDCS group. All values are normalized to the baseline measurement

Extracting the individual values of each subject at every time point in this cluster and plotting the difference in volumetric GM changes between tDCS and sham patients reveals that these dynamic longitudinal GM changes in the lingual gyrus effectively parallel the development of the clinical effect after tDCS treatment (see Fig. 4).

### Correlation of migraine days and GM changes

The associations between longitudinal GM changes in the lingual gyrus and the development of the number of migraine days after tDCS treatment did not show any significant correlation between these two parameters in this patient cohort.

## Discussion

This study investigated structural differences between EM and healthy controls and the impact of a 28-day treatment period with occipital tDCS on brain structure, evaluated approximately at 50 and 170 days after initiation of the treatment. The cross-sectional analysis of this study reveals two major findings. First, episodic migraine patients show distinct volumetric alterations in occipital cortical areas, located in the left lingual gyrus, when comparing them with a healthy control population. Second, these volumetric alterations are not related to the severity of migraine at baseline and the duration of the disease in this study cohort. Investigating these volumetric changes in episodic migraineurs longitudinally after a 28-day period of daily occipital tDCS treatment, we can draw two important conclusions. First, the observed cortical alterations between episodic migraineurs and healthy controls located in the left lingual gyrus are dynamic and show volumetric decreases over time in tDCS treated patients compared to sham treated patients. Second, these dynamic changes in the lingual gyrus are not correlated with the change in monthly migraine days in this study cohort.

Our observation of altered cortical structure in occipital areas is in line with the findings of a large study that compared migraineurs with aura (*n* = 333) and healthy controls and found increased cortical thickness in the left lingual gyrus [41]. Similarities between the study populations comprise the high percentage of migraineurs with aura in our study. Likewise, several previous studies could demonstrate altered cortical thickness and cortical volume in visual areas [42–47]. Some of these studies found decreases in cortical thickness respectively cortical volume of migraineurs, while in other studies migraineurs possessed increased cortical thickness respectively cortical volume compared to healthy controls. It should also be mentioned that some studies could not replicate these findings [48–52]. However, there are certain aspects to consider when comparing the results of these different studies. First, the cortical volume in VBM assessments is a mixed measure of cortical surface area, cortical folding as well as cortical thickness [53]. Second, migraine is a disorder with a great variance of clinical phenotypes and its structural alterations with respect to several parameters such as presence of aura, attack frequency, age, phase of the migraine cycle, the lateralization of migraine attacks and the presence of interictal photosensitivity [45, 46, 54–60]. These factors introduce further variability and thus limit the comparability among many studies. Nevertheless, the consistent observation of alterations in occipital areas suggests that this region might indeed be affected by structural changes in migraine.

There is strong evidence of the involvement of visual areas in the pathophysiology of migraine in ways of altered visual processing, which might explain the frequent presence of visual symptoms such as photophobia and visual aura [61]. Previous studies demonstrated altered cortical excitability [22, 23, 26] as well as functional changes including altered functional activity upon visual stimuli and functional connectivity in visual areas [62–66]. Furthermore, many studies found a strong link between cortical spreading depression, a wave of depolarization starting in the occipital lobe, and visual aura, as well as the involvement of cortical spreading depression in migraine attacks without aura [67–69]. The observed structural differences in visual areas might represent structural correlates of these functional changes that have been reported in migraineurs. However, the direction of the association between the functional and structural findings has yet to be elucidated. It is still unclear, whether these structural alterations represent a predisposition or abnormalities that lead to the development of migraine disease or if they occur because of recurring migraine attacks.

We did not detect any significant correlations of the observed structural cortical alterations with clinical baseline parameters representing disease severity such as the number of monthly migraine days and migraine intensity, and the number of years that the patients have suffered from this disease. One possible explanation is that the structural alterations do not depend on disease severity but instead represent an intrinsic trait that is not modified by the severity or duration of migraine. However, these findings should be interpreted with caution due to the relatively small size of the study population.

Tracking the observed structural differences over time after a 28-day treatment period with occipital tDCS, we observed a dynamic in the cortical volume that seems to parallel the change in disease severity represented by a reduction in monthly migraine days. Notably, these longitudinal changes were located in the same area as the observed structural differences between migraineurs and healthy controls, suggesting that these alterations are plastic and seem to respond to tDCS treatment. Whether these changes are mainly induced by the tDCS treatment or reflect the improvement of migraine symptoms still needs to be elucidated. Until today, only very few studies have assessed longitudinal structural changes in migraine populations [70]. These studies, however, could show longitudinal dynamic cortical volumetric changes over one respectively four years as well as changes during the migraine cycle, speaking to the potential for plasticity of these structural alterations in migraineurs [28, 29, 57]. Another study tracked the morphometric changes in chronic migraineurs following sphenopalatine ganglion block over six weeks and similarly to our results found a change in cortical and subcortical morphology with improving migraine symptoms [30].

This finding might have implications for migraine treatment and future interventional trials, as we could demonstrate that the cortical alterations are not permanent and fixed but rather plastic and can be modified by successful migraine treatment. Tracking the morphological cortical changes post-treatment might lead to the discovery of new biomarkers for the assessment of treatment response.

Although both, local cortical volume and monthly migraine days decreased after tDCS treatment compared to sham treated patients, we did not detect significant longitudinal correlations between these two measures. However, two important factors limit the interpretability of this assessment. First, the study population was relatively small as already mentioned above. Second, due to the exploratory nature of this study the structural MRI examinations were scheduled and performed at time points that missed the period of maximal clinical effect size. The first follow-up was performed just before the clinical effect had completely evolved (in T3 and T4) and the second follow-up after the effect had already vanished. These two factors might explain the lack of significant association between the structural and clinical changes and they should be considered in future studies.

Further limitations of this study are the remaining heterogeneity of the study population by including both patients with and without aura (i.e. no subgroup analysis for the effect tDCS on aura presence was possible, as the number of patients with and without aura was too low. Further, the aura type in patients with aura was not examined), a rather large spectrum of disease duration, and the lack of information about the timing of the MRI examinations during the patients’ migraine cycle, hence no correction for ictal/interictal variability was possible. Finally, we did not collect data on the usual headache lateralization and side. Thus, we do not know if any potential lateralization could be related to the observed dominant findings in the left hemisphere.

## Conclusions

Our results support the presence of structural changes in visual areas in episodic migraineurs. Whether these structural changes represent the substrate for migraine development or result due to recurring migraine attacks still needs to be elucidated. Furthermore, while the number of monthly migraine days decreased after tDCS treatment compared to sham treated patients, the cortical volume decreased as well. This paves the way for the development of new imaging biomarkers based on measuring structural volumetric changes for tracking the treatment effect of specific migraine therapies.

## Data Availability

The datasets presented in this article are not readily available due to Swiss law, the researchers must assess whether the use of the data and coded datasets are within the primary scope of the informed consent. Data is only available upon request and after the researchers have reviewed the purpose of the inquiry. Requests to access the datasets should be directed to Dr. Lars Michels, lars.michels@usz.ch.

## Abbreviations

CE: Cluster extent
CSF: Cerebrospinal fluid
DARTEL: Diffeomorphic Anatomical Registration using Exponentiated Lie algebra
EM: Episodic migraineur
FDR: False discovery rate
FUP: Follow-up
FWE: Family-wise error
GM: Grey matter
HADS-D: Hospital Anxiety and Depression Scale, Depression subscale
ICHD-3: International Classification of Headache Disorders, 3rd edition
MNI: Montreal Neurological Institute
MPRAGE: Magnetization prepared rapid gradient echo
MRI: Magnetic resonance imaging
MwA: Migraineur with aura
MwoA: Migraineur without aura
TBM: Tensor-based morphometry
tDCS: Transcranial direct current stimulation
TIV: Total intracranial volume
VBM: Voxel-based morphometry
WM: White matter
